# Hep-2–Adherent *Escherichia coli* Strains Associated with Acute Infantile Diarrhea, São Paulo, Brazil

**DOI:** 10.3201/eid0808.010492

**Published:** 2002-08

**Authors:** Isabel C. A. Scaletsky, Sandra H. Fabbricotti, Sueli O. C. Silva, Mauro B. Morais, Ulysses Fagundes-Neto

**Affiliations:** * Universidade Federal de São Paulo, Escola Paulista de Medicina, São Paulo, Brazil

**Keywords:** Adherent *Escherichia coli*, adherence, diarrhea

## Abstract

In this paired case-control study of infants with diarrhea in São Paulo, we examined the association between HEp-2–adherent *Escherichia coli* strains and diarrhea. We tested isolates from stool specimens of infants with diarrhea and matched controls in an HEp-2 cell adherence assay; we then hybridized isolates with DNA probes and identified enteropathogenic *E.* *coli* (EPEC), enteroaggregative *E. coli* (EAEC), and diffusely adherent *E. coli* (DAEC). From 100 patient-control pairs, we isolated 78 HEp-2–adherent strains; of these, 61 strains were single pathogens identified in stools of infants with diarrhea. While typical EPEC was significantly associated with diarrhea (p<0.001), EAEC was more frequently associated with diarrhea in clinical cases (20%) compared with healthy controls (3%) (p<0.001). Atypical EPEC, showing a localized adherence-like pattern, was also more common in patients than controls (p>0.1). DAEC was isolated with equal frequency from patients and controls (p>0.1).

HEp-2–adherent *Escherichia coli* strains that show localized adherence (LA), aggregative adherence (AA), diffuse adherence (DA), and localized adherence-like (LAL) patterns have been implicated as diarrheal pathogens (1). In a recent study, we reported the association of HEp-2–adherent *E. coli* strains, particularly those showing LAL pattern with diarrheal stools (2). HEp-2–adherent *E. coli* strains were also identified as the most important enteric pathotype in a paired case-control study of children with diarrhea <1 year of age in São Paulo, Brazil, from May to August 1985 (3). Enteropathogenic *E. coli* (EPEC) strains were most frequently identified (23%); patients and controls did not differ in the rate of isolation of diffusely adhering *E. coli* (DAEC) (31% and 32%, respectively) or enteroaggregative *E. coli* (EAEC) (10% and 8%, respectively).

The LA shown by typical EPEC is mediated by an inducible bundle-forming pilus, which correlates with the presence of a plasmid designated the EPEC adherence factor (EAF) plasmid (4,5). EPEC strains also cause attaching and effacing lesions on eukaryotic cells that involve a 94-kDa protein encoded by the chromosomal *eae* gene (6). The pathogenicity of EPEC strains has been demonstrated in human volunteers; the role of these strains in childhood diarrhea was confirmed in epidemiologic studies (1). Atypical EPEC strains do not carry the EAF plasmid and had an LAL pattern.

Two factors, F1845 and AIDA-I, were found to encode DA in DAEC (7,8). Several recent studies have implicated DAEC strains as agents of diarrhea (9,10), while other studies have not recovered DAEC strains more frequently from diarrheal patients than from asymptomatic controls (3,11). This association may be more frequent children >2 years of age (10).

The adherence of many EAEC strains requires the presence of a plasmid with localized genes coding for AA (1); a DNA fragment from an uncharacterized region of this plasmid was described as a specific EAEC probe (12). Epidemiologic studies have implicated EAEC as a cause of diarrhea in children in developing countries, and the pathogenic potential of EAEC in human infections was substantiated by challenge studies (1).

 In this study, we revisited the association between HEp-2–adherent strains and infants with diarrhea. We conducted a case-control study on *E. coli* isolates that were categorized as EPEC, EAEC, and DAEC by adherence tests and DNA probing. Our data suggest that EAEC may be a pathotype that is increasing in incidence as a cause of infantile diarrhea.

## Patients and Methods

### Patients

 At the Hospital São Paulo emergency room, fecal specimens were collected from infants (children <1 year of age) with acute diarrhea lasting <5 days and from individually age-matched control infants who visited the hospital at the same time for other reasons and had not had diarrhea during the previous 30 days; specimens were collected during July – August 1999. We collected patient-control pairs for the study until we had accumulated 100 pairs in which *E. coli* was detected in stools from both the patient and the control.

### Microbiologic Studies

*E. coli* strains were isolated on MacConkey plates. Four separate lactose-fermenting colonies, presumed to be *E. coli* by colony morphology, and two non-lactose-fermenting colonies of each distinct morphologic type were cultivated in commercial test systems (Probac do Brasil, São Paulo, Brazil) for biochemical confirmation of species or genus. *E. coli* colonies were subjected to slide agglutination with polyvalent and monovalent antisera (Probac do Brasil) against O antigens of EPEC serogroups and enterohemorrhagic *E. coli*. We tested the *E. coli* colonies by adhesion assay and hybridization with DNA probes (Table 1). *Salmonella* spp., *Shigella* spp., *Campylobacter* spp., *Yersinia enterocolitica*, and rotavirus were detected by standard methods (2).

**Table 1:** DNA probes identifying diarrheogenic *Escherichia coli* pathotypes^a^

*E. coli* pathotype	Specific for	DNA probe description	Reference
ETEC	LT enterotoxin	pCVD403 (1.3-kb *Bam*HI)	13
	STp enterotoxin	pCVD426 (157-bp *Pst*I)	
	STh enterotoxin	pCVD427 (216-bp *Eco*RI)	
EIEC	Invasion	pPS55 (2.5-kb *Hind*III)	14
EHEC	Adherence	pCVD419 (3.4-kb *Hind*III)	15
	Shiga toxin 1	pJN37-19 (1.142-kb *Bam*HI)	
	Shiga toxin 2	pNN110-18 (842-bp *Sma*I*-Pst*I)	
EPEC	EAF plasmid	pJPN16 (1-kb *Bam*HI*-Sal*I)	5
	*eae* gene	pCVD434 (1-kb *Sal*I*-Kpn*I)	6
DAEC	*daa*C *gene*	pSLM852 (390-bp *Pst*I)	7
	AIDA-I		8
EAEC	AA plasmid	pCVD432 (1-kb *Eco*RI*-Pst*I)	12

^a^ETEC, enterotoxigenic *E. coli*; EIEC, enteroinvasive *E. coli*; EHEC, enterohemorrhagic *E. coli*; EPEC, enteropathogenic *E. coli*; DAEC, diffusely adherent *E. coli*; EAEC, enteroaggregative *E. coli*; EAF, EPEC adherence factor; *eae*, encoding intimin, an outer membrane protein involved in the attaching and effacing lesions promoted by EPEC; *daa*C*,* associated with the biogenesis of F1845, a fimbrial adhesin involved in DA; AIDA-I, protein associated with the DA phenotype; AA, aggregative adherence plasmid.

 All *E. coli* isolates were characterized by the pattern of adherence to HEp-2 cells in the presence of D-mannose, as described by Scaletsky et al. (16). Monolayers were examined after 3 h of incubation. Briefly, monolayers of 10^5^ HEp-2 cells were grown in Dulbecco’s modified Eagle medium (DMEM) (Gibco-BRL, Gaithersburg, MD) containing 10% fetal bovine serum using 24-well plates (Becton, Dickinson and Company, Franklin Lakes, NJ). Bacterial strains were grown in 3 mL of Tryptic Soy Broth (Difco Laboratories, Detroit, MI) for 16 h –18 h at 37°C. Cell monolayers were infected with approximately 3 x 10^7^ bacteria (40 µL of bacterial cultures) added to 1 mL of DMEM and incubated at 37°C for 3 h. The infected monolayers were washed with sterile phosphate-buffered saline, fixed with methanol, stained with May-Grunwald-Giemsa stain, and examined under a microscope. When the adherence pattern was weak or negative, a new preparation was made and examined after a 6-h incubation period.

All *E. coli* isolates were screened by colony hybridization with DNA probes (Table 1). These probes were labeled by random primer extension kit (Rediprime II DNA Labelling System, Amersham Biosciences, Inc., Piscataway, NJ) with 50 µCi of [α-^32^P]dCTP. Colony blots were hybridized at 65°C overnight, washed with 0.1X SSC (1X SSC is 0.15 M NaCl plus 0.015 M sodium citrate) – 0.1% sodium dodecyl sulfate, and exposed to X-ray film overnight at -80°C.

 Data derived from infants with diarrhea and from control infants were compared by a two-tailed chi-square or Fisher’s exact test.

## Results

In total, we tested 402 and 430 *E. coli* colonies from 100 patients and 100 controls, respectively. We identified HEp-2–adherent strains in stool specimens from 61 infants with diarrhea. These strains were all isolated as the only pathogen; no mixed infections with a HEp-2–adherent strain and another pathogen (including enterotoxigenic *E. coli*, enteroinvasive *E. coli*, enterohemorrhagic *E. coli*, *Shigella* spp., *Salmonella* spp., *Campylobacter* spp., *Y. enterocolitica*, and rotavirus) were detected in any of the cases studied. Seventeen of the controls had HEp-2 adherent isolates in their stools (Table 2). Fifty-two of the 61 adherent strains were positive for DNA sequences for EPEC, EAEC, and DAEC strains. None of the nonadherent bacteria from patients or controls were positive in the hybridization assays.

**Table 2:** HEp-2 adherence of *Escherichia coli* strains isolated from infants with and without diarrhea

Adherence pattern, DNA probe^a^	No. of infected children		p value
	Patients^b^	Controls^b^	
Aggregative adherence (AA)	20	3	<0.001
AA^+^	17	2	<0.001
Localized adherence (LA)	17	0	<0.001
*eae*^+^, EAF^+^	17	0	<0.001
Localized adherence-like pattern (LAL)	6	2	0.279
*eae^+^*	6	2	0.279
Diffuse adherence (DA)	18	12	0.322
*daa*C^+^	12	8	0.480

^a^*eae*, gene encoding intimin, an outer membrane protein involved in the attaching of effacing lesions promoted by enteropathogenic *E. coli*; EAF, enteropathogenic *E. coli* adherence factor*; daa*C*,* gene associated with the biogenesis of F1845, a fimbrial adhesin involved in DA; +, positive. ^b^ n=100.

We observed four distinct patterns of adherence: LA occurred when the bacteria attached to localized areas of the HEp-2 cells in culture, forming distinct microcolonies after 3 h of incubation; DA occurred when bacteria adhered to the entire surface of the HEp-2 cells without formation of discrete microcolonies; AA was distinguished by prominent autoagglutination of the bacterial cells to each other on the surface of the cells, as well as those of glass or plastic containers; and LAL pattern, observed only in strains incubated for 6 h, was characterized by the formation of microcolonies or clusters less dense and compact than those displayed by typical LA-positive strains.

*E. coli* showing an AA pattern was more common in patients (20%) than in controls (3%) (p<0.001) and was detected more frequently than EPEC (17%) in patients. Nineteen (83%) of 23 *E. coli* isolates with the AA pattern hybridized with the AA probe. Of the EAEC isolates, two from patients belonged to the classic EPEC O serogroup (O44 and O78); one from a control belonged to O126.

Strains with LA were significantly associated with diarrhea (17% vs. 0%; p<0.001). Typical EPEC was analyzed on the basis of *eae* and EAF-positive probes; all *E. coli* isolates showing the LA pattern were hybridized with both probes. By using the *eae* DNA probe, which is specific for atypical EPEC, we found eight strains to be positive. These eight atypical EPEC strains, which showed an LAL pattern, were isolated from six patients (6%) and two controls (2%). Of the 17 typical EPEC isolates, 15 were from classic EPEC O serogroups (4 from O55, 1 from O86, 2 from O111, and 8 from O119). Of the eight atypical EPEC isolates, seven belonged to classic serogroups (one from O26, two from O111, two from O127, and two from O128).

We found a high rate of isolation for *E. coli* that adhered with a DA pattern; however, rates were similar in patients (18%) and controls (12%). Of the 30 DAEC isolates, 20 hybridized with the *daa*C probe and none with the AIDA-I probe. Only two isolates belonged to classic serogroups (O15 and O158).

## Discussion

While EPEC has long been considered the dominant *E. coli* pathogen in São Paulo, an epidemiologic association between EAEC and acute diarrhea has not been found in Brazil until now. LA-positive EPEC has been shown to be associated with infantile diarrhea in several paired case-control studies of children <1 year of age in São Paulo (5,12). In our study, those strains were significantly more often isolated from diarrheal stools (p<0.001), demonstrating that EPEC continues to be an important cause of diarrheal disease in São Paulo. LA production was associated with EPEC O serogroups, as described by other researchers (2,17).

*E. coli* strains that exhibited AA and were hybridized with the AA probe were strongly associated with enteric diseases in São Paulo. Moreover, the phenotypic and genotypic approaches for the identification of EAEC strains gave almost similar results: 17/20 EAEC strains from children with diarrhea and 2/3 EAEC strains from the control group hybridized with the AA probe. In an epidemiologic study in another region of northeast Brazil, EAEC probe-positive and probe-negative strains were more likely to be associated with persistent diarrhea (18,19). Our results demonstrate that 83% of EAEC strains that were probe positive were associated with diarrhea in infants in São Paulo. We found some AA isolates belonging to the EPEC O serogroups, a finding that has been demonstrated by other authors (1). The present study is the first to show high prevalence of EAEC; in the last 25 years, EPEC strains have been prevalent in Brazil.

The pathogenic role of *E. coli* showing a DA pattern (DAEC) in the etiology of diarrheal disease is controversial (3,9–11). We found no correlation between DAEC strains and diarrhea; our results agree with those of epidemiologic studies in Australia and France (10,11), namely, that DAEC may be important diarrheal pathogens in children >1 year of age. The *daa*C probe did not show a good correlation with the DA phenotype in our study.

This study suggests for the first time that EAEC may have become a major etiologic agent of acute diarrhea in São Paulo. Further studies are needed to investigate the pathogenesis of the EAEC strains isolated in this study.
